# Globalization and life lost due to tuberculosis: evidence from a multi-country study

**DOI:** 10.12688/f1000research.74445.1

**Published:** 2021-12-07

**Authors:** Shyamkumar Sriram, Muayad Albadrani

**Affiliations:** 1 Johns Hopkins, Johns Hopkins Bloomberg School of Public Health, Baltimore, MD, 21205, USA; 2 Taibah University, Taibah University, Medina, Medina, Saudi Arabia

**Keywords:** Globalization, tuberculosis, Years of Life Lost, mortality

## Abstract

**Background**: Tuberculosis affects around 30% of the population of the world. Tuberculosis causes an increase in early mortality and thus has the potential to increase the number of years of life lost. Globalization directly or indirectly by affecting the factors that increase the susceptibility for tuberculosis infection has the potential to increase the spread and mortality due to tuberculosis. This study assessed the causal link between globalization and the years of life lost due to tuberculosis.

**Methods**: Data from the Demographic and Health Survey (DHS) and World Bank for 2004 and 2005 were used for a number of covariates and possible mediators. Data from the Institute of Health Metrics and Evaluation (IHME) were used for the outcome variable and important globalization indicators. The primary health outcome that was studied is tuberculosis and the measure that was used to quantify tuberculosis mortality is the years of life lost (YLL). Path analysis was used.

**Results**: The main independent variables of economic and social integration were not statistically significant. For every unit increase in the proportion of people that were using treated drinking water, there was a -0.0002 decrease in the YLL due to tuberculosis. For every unit increase in the proportion of people with earth floor, there was a 0.0002 units increase in YLL due to tuberculosis. For every unit increase in the proportion of people living using clean fuel, there was a 0.0004 decrease in the YLL due to tuberculosis.

**Conclusions:** Social and economic globalization have no effect on the years of life lost due to tuberculosis, highlighting that globalization actually does not contribute to tuberculosis mortality. However, improving other important determinants such as sanitation, providing safe drinking water and clean households will reduce the mortality due to tuberculosis, highlighting the need to invest in them.

## Background

Tuberculosis (TB) is a disease that is caused by the bacteria, Mycobacterium tuberculosis. Tuberculosis affects around 30% of the population of the world. ^1^ Incidence of tuberculosis is around nine million new cases every year. ^1^ Studies globally show that tuberculosis causes an increase in early mortality and thus has the potential to increase the number of years of life lost. Annual mortality due to tuberculosis is around one and half million. ^1^ The transmission of the disease is predominantly airborne in most of the cases and exposure to the disease agent is needed for any person to develop the infection or the disease. ^2^ However, there are many individual, household and environmental factors that increase the susceptibility of the individual to develop the infection. ^3^ Globally, the disease which had been controlled over the past two decades has shown to be reemerging in a number of countries, especially in the developing world. ^4^^–^^6^ Globalization, specifically the negative effects and externalities of globalization have shown to increase the spread of a number of infectious diseases. Some studies have explored the effect of globalization on tuberculosis spread and found that globalization increases the spread of tuberculosis, especially in countries with poverty and poor health systems. ^7^ Evidence also shows that globalization has also been found to have a negative effect on poverty specifically by increasing poverty for certain underprivileged sections of the population. ^8^ Some of the factors which increase the susceptibility of an individual to develop tuberculosis such as living in over-crowded houses, having poor sanitation facilities, poor housing conditions, and the consumption of unsafe water are all directly related to the availability of household resources and poverty. ^7^^,^^9^ Thus, globalization, by directly or indirectly affecting the factors that increase the susceptibility for tuberculosis infection, has the potential to increase the spread and mortality due to tuberculosis and in turn increasing the number of years of life lost.

There are many aspects and components of globalization. There is a constant movement of people across the globe (social globalization) and the flow of capital across countries (economic globalization). ^10^ Both social and economic components of globalization have the potential to increase the spread of tuberculosis either directly by increased movement and contact of people or by causing changes in the economy which affects the factors that increase the susceptibility to develop tuberculosis. Evidence shows that in some populations globalization increased their economic conditions, while in others globalization reduced their economic status leading to an increase in poverty, unemployment, and unsanitary living conditions. ^11^^,^^12^ There is only scarce literature linking globalization and tuberculosis. ^2^^,^^3^^,^^13^^–^^15^ Current available literature on globalization and tuberculosis is able to only correlate the increase in globalization over the past decade and the increase in burden of tuberculosis in specific countries of the world. These studies failed to prove conclusively that globalization by itself leads to changes in the spread of tuberculosis, although some patterns were observed. Also, the studies on globalization are more prone to confounding because of many unforeseen factors. Currently evidence on the effect of globalization on the mortality and years of life lost due to tuberculosis is lacking. This study aims to address this gap in literature through the empirical use of secondary data that is available from several international agencies to quantify and assess the causal link between globalization and the years of life lost due to tuberculosis.

## Methods

### Ethical approval

The secondary datasets used in this study are available in the public domain after all individual level identification variables were removed. It was not possible to identify the residence of any of the households as well. Therefore, ethical approval was not required for the study.

### Consent for participation/publication

Not applicable

### Study population

This study examined the population globally representing 40 countries from every continent and every WHO region in the world. The specific age-group that was studied is the age group of 15 years and above. The primary health outcome that was studied is tuberculosis and the measure that was used to quantify tuberculosis mortality was the years of life lost (YLL). YLL for TB for 2010 was used in the study. Tuberculosis has an incubation period of a few months to about two years from exposure to the development of disease. The independent variables was studied for 2005. This five year gap not only provided the time for the disease to develop from exposure but also the period when there was a shift in the independent variables and the impact of those variables on the population health outcome, TB.

### Data

The DHS Program STATcompiler was used to extract the data from the DHS and World Bank. Data on the proportion of households with poor household sanitation, open defecation, safe drinking water, smoking cigarettes, literacy, type of cooking fuel, BCG vaccination, overcrowding, and unclean floors were obtained from the Demographic and Health Survey (DHS) for years 2004 and 2005. Data on the Poverty headcount and GNI per capita were obtained from the World Bank for the years 2004 and 2005. Data were assessed for a number of covariates and possible mediators. Data from the Institute of Health Metrics and Evaluation (IHME) were used for the outcome variable (Years of Life Lost due to Tuberculosis) and important globalization indicators (Economic globalization index and Social globalization index) for the year 2010 were used. The time-period for tuberculosis development varies. Tuberculosis has an incubation period of a few months to about two years from exposure to the development of disease. The effect of the key independent variable and other variables on the development of tuberculosis can be studied only if they are examined for at least three to five years before 2010 (the year when tuberculosis outcome is studied).

### Analytic framework

**Analytic framework.**
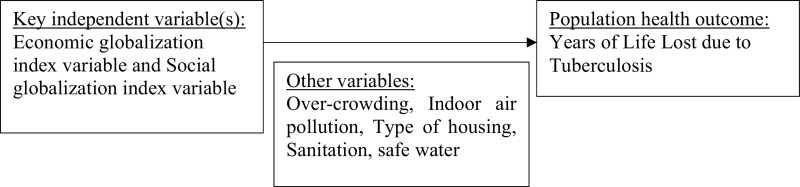


### Variables of interest/key variables

The key dependent variable was the Years of Life Lost (YLL) due to tuberculosis in 40 countries. The main independent variables included the variables of globalization index namely the variable for economic integration and social integration. The possible mediators included the variables for sanitation, clean drinking water, indoor air pollution (clean cooking fuel), and over-crowding.

### Statistical analysis

Descriptive analysis was be used to describe the trends in the data. Path analysis was used to study both the effect of the various independent variables on the outcome variable and also to study the effect of the globalization index variables (economic and social integration) on the mediator variables. Path analysis helped in understanding the directed dependencies among the different variables. Statistical software STATA (version 17) was be used for the data analysis.

## Results

### Descriptive statistics

The average overall proportion of population having improved household sanitation facility was 49.10% as shown in Table 1. Table 2 shows the descriptive statistics by country. In Jordan (99.6%), the highest proportion of population had improved sanitation facility while Madagascar (4.2%) had the lowest sanitation facility. The average overall proportion of people living in households treating drinking water was 66.77%. Egypt (95.5%) had the highest proportion of population living in households that were treating drinking water. The average overall proportion of people using clean fuel for cooking was 25.44%. The proportion varied from 99.9% in Jordan to none of the population having clean cooking fuel in Sierra Leone. The average overall proportion of population in households with seven or more persons sleeping per room was 2.50%. India had the highest proportion of overcrowding in houses, while Indonesia had the lowest overcrowding among the list of selected countries. The average overall proportion of people living in houses with earth/sand floors was 37.70%. Niger around 87.2% of the houses having sand floors, while Jordan had only 0.1% of the houses with sand floors. The average years of life lost due to tuberculosis was 0.0323 years with Indonesia facing the highest years of life lost due to TB, while counties like Guyana, Kenya, Peru facing the lowest years of life lost due to TB. The average economic integration score was 2.97. Uganda (4.92) had the highest economic integration, while Haiti (0.34) had the lowest economic integration. The average social integration score was 1.09. Uganda (6.90) had the highest social integration, while Nepal (0.03) had the lowest social integration.

**Table 1.** Descriptive statistics for whole sample.

Variables	Definition	Mean (SD)
Prop_Pop_Impro_Sanitation	Proportion of population having improved household sanitation facility	49.10 (27.60)
Water_treated	Proportion of people living in households treating drinking water	66.77 (24.72)
CookingFuel	Proportion of people using clean fuel for cooking	25.44 (28.78)
rooms_7more	Proportion of population in households with 7+ persons sleeping per room	2.50 (1.66)
Earth_sandfloors	Proportion of people living in houses with earth/sand floors	37.70 (25.33)
YLL Tuberculosis	Average years of life lost due to Tuberculosis	0.0323 (0.0314)
Economic integration	Economic integration	2.97 (1.15)
Social integration	Social integration	1.09 (1.03)

**Table 2.** Descriptive statistics by country.

S. No	Country	YLL-TB	Economic integration	Social integration	Sanitation	Treated water	Sand floors
1	Bangladesh	0.0424	2.56	1.03	24.30	92.50	79.60
2	Benin	0.0310	0.59	0.81	17.10	93.80	41.00
3	Bolivia	0.0323	4.32	1.10	11.30	14.60	29.10
4	Brazil	0.0051	3.50	1.54	49.40	66.50	6.40
5	Cameroon	0.0202	2.50	0.99	35.00	11.30	51.10
6	CAR	0.0423	2.02	0.37	14.40	23.80	81.60
7	Colombia	0.0054	3.61	1.39	87.20	80.90	8.50
8	DRC	0.0652	2.24	0.21	40.10	71.50	74.70
9	Egypt	0.0027	3.41	1.32	99.50	95.50	11.90
10	Gabon	0.0185	2.62	0.31	46.60	32.30	21.40
11	Ghana	0.0217	2.78	1.40	66.40	89.80	15.10
12	Guatemala	0.0074	3.89	1.45	41.20	56.40	41.70
13	Guyana	0.0103	3.53	0.72	92.40	51.90	2.90
14	Haiti	0.0125	0.34	1.07	29.80	66.90	38.50
15	Honduras	0.0059	3.85	1.20	64.00	55.00	26.40
16	India	0.0527	2.26	1.01	39.70	66.80	33.10
17	Indonesia	0.1273	3.85	0.96	52.00	6.30	11.40
18	Jordan	0.0020	3.93	1.00	99.60	78.50	0.10
19	Kenya	0.0393	3.33	0.81	44.60	56.30	37.70
20	Madagascar	0.0275	1.56	0.76	4.20	49.30	15.10
21	Malawi	0.0356	2.61	0.88	4.80	34.90	77.60
22	Mali	0.0390	2.00	0.51	21.30	66.10	58.10
23	Mauritius	0.0275	3.89	1.70	22.40	32.50	62.70
24	Namibia	0.0451	2.99	1.22	42.10	90.40	46.40
25	Nepal	0.0658	2.61	0.03	36.50	86.50	69.30
26	Nicaragua	0.0129	4.66	1.18	33.70	34.30	45.50
27	Niger	0.0493	1.86	0.70	9.20	69.00	87.20
28	Nigeria	0.0317	2.72	0.16	53.00	84.30	35.90
29	Pakistan	0.0528	1.58	1.12	53.20	90.00	49.60
30	Peru	0.0230	4.22	1.11	67.00	12.60	40.10
31	Philippines	0.0490	3.60	1.16	86.30	69.10	8.60
32	Rwanda	0.0592	1.33	0.33	55.60	56.10	83.20
33	Senegal	0.0384	3.00	1.02	80.50	35.60	26.10
34	Sierra Leone	0.0443	2.10	0.13	44.10	89.00	56.30
35	Togo	0.0327	0.98	0.87	29.30	37.30	27.80
36	Turkey	0.0040	4.04	1.65	99.10	23.50	3.80
37	Uganda	0.0582	4.92	6.90	24.30	60.70	39.10
38	Ukraine	0.0162	3.77	0.74	98.50	15.80	0.20
39	Zambia	0.0376	4.62	1.19	37.40	63.20	59.30
40	Zimbabwe	0.0460	4.62	1.19	56.40	25.10	16.70

### Regression analysis

A path analysis model with an OLS regression component was used to study the effect of the main independent variables of globalization (economic and social globalization) and other covariates and potential mediating variables on the outcome variable of years of life lost due to tuberculosis. The results of the regression analysis are shown in Table 3. The main independent variables of economic and social integration were not statistically significant showing that they have no significant effect on the years of life lost (YLL) due to tuberculosis. Other possible mediator variables such as proportion of population having improved household sanitation facility did not have any statistically significant effect on the YLL due to tuberculosis. The variables of proportion of people living in households treating drinking water, the variable proportion of people using clean fuel for cooking and the proportion of people living in houses with earth/sand floors were statistically significant. For every unit increase in the proportion of people living in households that were using treated drinking water, there was a −0.0002 decrease in the YLL due to tuberculosis. For every unit increase in the proportion of people living in houses with earth or sand floor, there was 0.0002 units increase in the YLL due to tuberculosis. For every unit increase in the proportion of people living using clean fuel for cooking, there was a 0.0004 decrease in the YLL due to tuberculosis. The results of the path regression analysis show that economic integration had a positive impact on sanitation and a negative effect on water treatment and the proportion of households with sand floors. Social integration on the other hand seemed to have a negative impact on sanitation, while having a positive effect on the proportion of households that were treating water.

**Table 3.** Path regression analysis on the factors affecting Years of Life Lost due to TB.

Characteristics	Coefficient	Standard error	P value
**Mean years of life lost due to TB**			
Economic integration	0.0035789	0.0019183	0.06
Social integration	0.0004549	0.0013553	0.73
Proportion of population having improved household sanitation facility	0.0001243	0.0001231	0.31
Proportion of people living in households treating drinking water	−0.0002498	0.0000757	<0.01
Proportion of people using clean fuel for cooking	−0.0004893	0.0000879	<0.01
Proportion of people living in houses with earth/sand floors	0.0002075	0.0000905	0.02
Constant	0.040401	0.0074816	<0.01
**Proportion of population having improved household sanitation facility**			
Economic integration	14.48399	0.7772967	<0.01
Social integration	−8.025419	0.7054411	<0.01
Constant	14.89532	2.140847	<0.01
**Proportion of people living in households treating drinking water**			
Economic integration	−8.65949	0.7769014	<0.01
Social integration	1.54382	0.7050823	0.02
Constant	90.4312	2.139758	<0.01
**Proportion of people living in houses with earth/sand floors**			
Economic integration	−5.542206	0.8563732	<0.01
Social integration	0.9719376	0.7772075	0.21
Constant	52.56139	2.358641	<0.01

## Discussion

The results of the study show that there is no effect of social and economic globalization on the years of life lost due to tuberculosis. Literature shows that there are no other studies that have explored this specific relationship of globalization in the context of years of life lost due to tuberculosis. However, there are other studies which have explored similar relationships between globalization and other diseases and outcome measures. ^10^ For example, a study analyzed the effect of social and economic globalization on obesity and overweight and concluded that the different components of globalization increased the propensity of the individual to become overweight, especially among women. ^10^ However, in this research, no such relationships could be established in the context of globalization and tuberculosis. This study shows that sanitary conditions in the household are not statistically associated with the years of life lost due to tuberculosis. However, other studies have shown that living in a household with good sanitary conditions not only decreased the propensity of developing tuberculosis but also reduced the mortality due to tuberculosis. ^9^^,^^16^^,^^17^ This research also shows that people who are living in households that are consuming treated drinking water had lower years of life lost due to tuberculosis. This is consistent with findings in literature which show that households that consume boiled and treated safe drinking water had lower risk of developing tuberculosis and also associated lower mortality due to tuberculosis. ^16^^,^^18^^–^^20^ The people who live in houses that have dusty environment with earth or sand floors have been found to have higher mortality due to tuberculosis and thus higher years of lost. This aligns with the findings from literature which show that living in an unclean and dusty environment increases the susceptibility of developing tuberculosis and also increasing the progression and severity of the disease leading to increased mortality. ^16^^,^^21^ Houses with dirty floors and dust serve as a vehicle for transmission for tuberculosis, especially if the patient removes saliva or phlegm on the floor of the house. In addition, evidence shows that there is a higher probability of reactivation of tuberculosis infection among individuals who live in insanitary and unclean household conditions. ^22^ Indoor air pollution has shown to be an important predisposing and enabling factor for tuberculosis. ^23^^,^^24^ Usage of unclean cooking fuel which is rampant in developing countries is one of the important causes globally for indoor air pollution. ^25^^,^^26^ This study also supports this finding. Households that use clean cooking fuel has been found to have lower years of life lost due to tuberculosis compared to households that used unclean fuel for cooking. This study shows that economic integration has a positive effect on sanitation. There is no specific literature which relates economic globalization with sanitation however some evidence shows that globalization increases household wealth in some populations, and this is directly proportional to increased levels of sanitation. ^15^ The negative relationship between economic globalization and poor access to safe drinking water could not be supported by any evidence from literature. In this study, social integration has a negative impact on household sanitation. Although no specific evidence linking social globalization and household sanitation could be found, some evidence from literature show that globalization has the potential to push certain groups of the population into poverty which reduces their financial capacity to maintain proper hygiene and sanitation in the household. ^8^^,^^11^^,^^12^^,^^27^^–^^30^

## Conclusions

This study provides vital analysis into the different aspects of globalization and their effect on tuberculosis spread in many countries across different continents. This will help in global policy making regarding infectious disease spread. Social and economic globalization have no effect on the years of life lost due to tuberculosis, highlighting that globalization actually does not contribute to tuberculosis mortality. This will help in clearing the common myth that globalization is one of the main reasons for tuberculosis spread across national borders. However, improving other important determinants such as sanitation, providing safe drinking water and clean households will reduce the mortality due to tuberculosis, highlighting the need to invest in them. In conclusion this research could not find any relationship between globalization and the years of life lost due to tuberculosis. More research is needed to understand the mechanism in which social and economic globalization influence tuberculosis specifically the years of life lost due to tuberculosis.

### Strengths and limitations

The main strengths of this study include the use of secondary data from multiple international agencies which have a reliable and robust method of data collection and adequate data quality control mechanisms in place. The study also considers the adequate time lag between the independent variables (exposures) and the health outcome to develop. The main limitations of this study also arise from the use of secondary data. Any study that uses secondary data suffers from this limitation, i.e., the study becomes limited by the data collected and survey methodology used. The contents and the questions asked in the survey limits the extent of our research questions to fit the data that is available for analysis. Also, cross-sectional data has been used for the study and thus no follow-up over time has been done. This cross-sectional nature of the data creates an important limitation that it allows to study only the association and not actual causation of mortality due to tuberculosis because of globalization to be established. Cross-sectional data cannot infer causal association mainly because temporality is not known and thus cannot assess the change in outcomes over time. The limitations of the model include the use of path regression analysis. Although the OLS component of the path regression model can be used to establish association between the outcome variable and the various independent variables, but path analysis is not a causality test so the causal associations of the potential mediating variables cannot be ascertained. ^31^

## Data availability

The DHS Program STATcompiler was used to extract the data from the DHS and World Bank. Data on the proportion of households with poor household sanitation, open defecation, safe drinking water, smoking cigarettes, literacy, type of cooking fuel, BCG vaccination, overcrowding, and unclean floors were obtained from the Demographic and Health Survey (DHS) for years 2004 and 2005. Access to the dataset requires registration and is granted only for legitimate research purposes. A guide for how to apply for dataset access is available at: https://dhsprogram.com/data/Access-Instructions.cfm. Data on the Poverty headcount and GNI per capita were obtained from the World Bank for the years 2004 and 2005. World Bank data is available for open access. Poverty headcount data is available at: https://data.worldbank.org/indicator/SI.POV.DDAY and GNI per capita is available at: https://data.worldbank.org/indicator/NY.GNP.PCAP.CD. Data from the Institute of Health Metrics and Evaluation (IHME) were used for the outcome variable (Years of Life Lost due to Tuberculosis) and important globalization indicators (Economic integration and integration) for the year 2010 was used. Data can be obtained from the IHME website: http://www.healthdata.org/.
