# Supplementary material for: Globalization and life lost due to tuberculosis: evidence from a multi-country study
Source: F1000Res. 2021 Dec 7;10:1251. [Version 1] doi: 10.12688/f1000research.74445.1 (PMC8977916; doi:10.12688/f1000research.74445.1)
Supplement: Peer review history

# Peer review: Reviewer response for version 1

**DOI:** 10.5256/f1000research.78203.r119880

**Published:** 2022-01-18

**Authors:** Arutselvi Devarajan

**Affiliations:** 1 Department of Epidemiology, M.V. Hospital for Diabetes and Prof. M. Viswanathan Diabetes Research Centre (WHO Collaborating Centre for Research, Education and Training in Diabetes) (IDF Centre for Excellence in Diabetes Care), Chennai, India

This paper explored an important aspect of the public health issue of TB and its association with globalization.

 I have some suggestions for the authors:

 The nature of data was cited as the reason for not being able to completely explain the causal link, I suggest the authors mention only association (as the data may exhibit some correlation but not causation) instead of the "causal link" in the objective. 

 Although the current introduction is good, it would be better if there are more indirect indicators or covariates that affect tuberculosis incidence. 

 The methods section is good and elaborate. The aspects of globalization - economic and social, and other aspects of globalization could also be considered in this research or for future research. 

 The main outcome variable is Years of Life Lost due to tuberculosis. It would be much better if disability-adjusted life years could have been used in future papers to expand this research.

Is the work clearly and accurately presented and does it cite the current literature?

Yes

If applicable, is the statistical analysis and its interpretation appropriate?

Yes

Are all the source data underlying the results available to ensure full reproducibility?

Yes

Is the study design appropriate and is the work technically sound?

Yes

Are the conclusions drawn adequately supported by the results?

Yes

Are sufficient details of methods and analysis provided to allow replication by others?

Yes

Reviewer Expertise:

Interaction of Tuberculosis and Diabetes, Health Systems Research,  Diabetic epidemiology, Pscyhosocial Research

I confirm that I have read this submission and believe that I have an appropriate level of expertise to confirm that it is of an acceptable scientific standard.

# Peer review: Reviewer response for version 1

**DOI:** 10.5256/f1000research.78203.r119876

**Published:** 2022-01-17

**Authors:** Mohamed Adil AA

**Affiliations:** 1 Vinayaga Mission Research Foundation, Chennai, Tamil Nadu, India

The article highlights important areas in the arena of globalization and the spread of infectious diseases.

 The article particularly looks into data from a number of countries globally, thus increasing the validity and reliability of the study across continents and also globally. 

 Could this study be replicated by using longitudinal data to establish causality and stronger inferences?

 Do the path regression results provide more robust results than OLS analysis?

 What was the main logic in choosing only a specific set of covariates and not all the possible covariates for tuberculosis? 

 This a good study and will help in addressing many lacunae in the area of global health research.

Is the work clearly and accurately presented and does it cite the current literature?

Yes

If applicable, is the statistical analysis and its interpretation appropriate?

Yes

Are all the source data underlying the results available to ensure full reproducibility?

Yes

Is the study design appropriate and is the work technically sound?

Yes

Are the conclusions drawn adequately supported by the results?

Yes

Are sufficient details of methods and analysis provided to allow replication by others?

Yes

Reviewer Expertise:

Infectious Diseases, Biotechnology, Disease Transmission Dynamics

I confirm that I have read this submission and believe that I have an appropriate level of expertise to confirm that it is of an acceptable scientific standard.

# Peer review: Reviewer response for version 1

**DOI:** 10.5256/f1000research.78203.r102228

**Published:** 2022-01-14

**Authors:** Setya Haksama

**Affiliations:** 1 Department of Health Administration and Policy, Public Health Faculty, Universitas Airlangga, Surabaya, Indonesia

1. All variables should be written clearly and systematically, first the independent variables should be described, then the dependent variables should be described.

 2. Resources of data from World Bank was too old.

 3. No data was obtained from 40 countries measured in relation to this research, there should be a ranking for each country that can indicate which countries have good scores and which countries have low scores.

Is the work clearly and accurately presented and does it cite the current literature?

Yes

If applicable, is the statistical analysis and its interpretation appropriate?

Partly

Are all the source data underlying the results available to ensure full reproducibility?

Yes

Is the study design appropriate and is the work technically sound?

Yes

Are the conclusions drawn adequately supported by the results?

Partly

Are sufficient details of methods and analysis provided to allow replication by others?

Partly

Reviewer Expertise:

Health Policy, Administration, and Disaster

I confirm that I have read this submission and believe that I have an appropriate level of expertise to confirm that it is of an acceptable scientific standard, however I have significant reservations, as outlined above.

# Response

**Published:** 2022-01-14

**Authors:** Shyamkumar Sriram

**Affiliations:** University of South Carolina, USA

Multiple data sources from multiple agencies were used. Data from the World Bank for specific years was used to match the data availability from other agencies. Data could not be obtained for the specific years for some of the countries. So they were not used for the research. Different variables have been adequately described in the paper.
